# Supplementary material for: Expanding the genetic architecture of nicotine dependence and its shared genetics with multiple traits
Source: Nat Commun. 2020 Nov 3;11:5562. doi: 10.1038/s41467-020-19265-z (PMC7642344; doi:10.1038/s41467-020-19265-z)
Supplement: Supplementary file 3 — Description of Additional Supplementary Data [file 41467_2020_19265_MOESM3_ESM.pdf]

Description of Additional Supplementary Files

File Name: Supplementary Data 1.
Description: Genome-wide significant single nucleotide polymorphism (SNP) and insertion/deletion (indel) associations from the cross-ancestry genome-wide association study (GWAS) meta-analysis (total N=58,000). The SNPs and indels span five loci: chromosomes 5q34, 7q21, 9q34, 15q25, and 20q13. “Direction” indicates the association of the “Allele1” allele, corresponding to the “Effect” (β coefficient, with + corresponding to increased risk and – corresponding to decreased risk of nicotine dependence), across the 23 studies. Nominal p-values were calculated by the METAL software package, which uses a two-sided Z-test.

File Name: Supplementary Data 2.
Description: Genome-wide H-MAGMA results using the nicotine dependence GWAS metaanalysis of European ancestry participants in the iNDiGO consortium with reference to chromatin interaction maps from fetal brain tissue. Results are sorted by the H-MAGMA F-test p-value.

File name: Supplementary Data 3.
Description: Genome-wide H-MAGMA results using the nicotine dependence GWAS metaanalysis of European ancestry participants in the iNDiGO consortium with reference to chromatin interaction maps from adult brain tissue. Results are sorted by the H-MAGMA F-test p-value.

File Name: Supplementary Data 4.
Description: Genome-wide Summary-MultiXcan (S-MultiXcan) results from the European ancestry-specific nicotine dependence GWAS meta-analysis summary statistics with reference to imputed genetically driven gene expression across the 13 adult brain tissues in GTEx. S-MultiXcan provides gene-level association results based on aggregating cis-eQTL evidence across multiple tissues, while also presenting gene-based results from the best and worst single-tissue models. Results are sorted by the multi-tissue F-test p-value.

File Name: Supplementary Data 5.
Description: Tissues and cell types evaluated for shared genetics with nicotine dependence (ND) using stratified linkage disequilibrium (LD) score regression, as applied to specifically expressed genes (LDSC-SEG). Tissues and cell types are sorted by data origin (RNA-sequencing in the GenotypeTissue Expression [GTEx] project or array-based in Gene Expression Omnibus [GEO]) and then by pvalue. Tissues/cell types that have statistically significant correlations with ND, as determined by Bonferroni correction (α=0.05/205 phenotypes, 1 degree of freedom Chi-square test, P < 2.4×10-4), are bolded.

File Name:Supplementary Data 6.
Description: Look-up of genome-wide significant single nucleotide polymorphisms (SNPs) in the GWAS and Sequencing Consortium of Alcohol and Nicotine use (GSCAN) consortium for association with nicotine dependence (ND) in our cross-ancestry GWAS meta-analysis. Results are sorted by GSCAN smoking phenotype in descending|rg|with ND (cigarettes per day [rg=0.95], age at initiation [rg=-0.55], cessation [current vs. former smoking, rg=0.51], and initiation [ever vs. never smoking, rg=0.40], shown in alternating grey shading) and then by FTND GWAS meta-analysis p-value (calculated by the METAL software package, which uses a two-sided Z-test). For SNPs implicated at genome-wide significance for more than one phenotype in GSCAN, the results from the phenotype with the smallest p-value (calculated by the Score test implemented in the RVTESTS software package) are presented. β estimates correspond to the effect alleles. No SNPs from novel loci for ND surpassed Bonferroni correction for multiple testing (P
