# Supplementary material for: Expanding the genetic architecture of nicotine dependence and its shared genetics with multiple traits
Source: Nat Commun. 2020 Nov 3;11:5562. doi: 10.1038/s41467-020-19265-z (PMC7642344; doi:10.1038/s41467-020-19265-z)
Supplement: Supplementary file 10 — Reporting Summary [file 41467_2020_19265_MOESM10_ESM.pdf]

nature research

Corresponding author(s): Dana Hancock

Last updated by author(s): Sep 11, 2020

Reporting Summary

Nature Research wishes to improve the reproducibility of the work that we publish. This form provides structure for consistency and transparency in reporting. For further information on Nature Research policies, see our Editorial Policies and the Editorial Policy Checklist.

Statistics

For all statistical analyses, confirm that the following items are present in the figure legend, table legend, main text, or Methods section.

n/a	Confirmed	
☐	☒	The exact sample size (n) for each experimental group/condition, given as a discrete number and unit of measurement
☐	☒	A statement on whether measurements were taken from distinct samples or whether the same sample was measured repeatedly
☐	☒	The statistical test(s) used AND whether they are one- or two-sided<br>*Only common tests should be described solely by name; describe more complex techniques in the Methods section.*
☐	☒	A description of all covariates tested
☐	☒	A description of any assumptions or corrections, such as tests of normality and adjustment for multiple comparisons
☐	☒	A full description of the statistical parameters including central tendency (e.g. means) or other basic estimates (e.g. regression coefficient) AND variation (e.g. standard deviation) or associated estimates of uncertainty (e.g. confidence intervals)
☐	☒	For null hypothesis testing, the test statistic (e.g. F, t, r) with confidence intervals, effect sizes, degrees of freedom and P value noted<br>*Give P values as exact values whenever suitable.*
☒	☐	For Bayesian analysis, information on the choice of priors and Markov chain Monte Carlo settings
☒	☐	For hierarchical and complex designs, identification of the appropriate level for tests and full reporting of outcomes
☐	☒	Estimates of effect sizes (e.g. Cohen's d, Pearson's r), indicating how they were calculated

Our web collection on statistics for biologists contains articles on many of the points above.

Software and code

Policy information about availability of computer code

Data collection

No software was used. Primary data collection was not done in this study.

Data analysis

The following publicly available software were used in the current study:
METAL (version 2011-03-25), http://csg.sph.umich.edu/abecasis/metal/download/
LocusZoom (version 1.3), http://csg.sph.umich.edu/locuszoom/download/
LDSC (version 1.0.0; git commit hash 89c13a7e5f933a84e839c4069228dbdcfc539c5d), https://github.com/bulik/ldsc
GCTA (versions 1.90-1.92), https://cnsgenomics.com/software/gcta/#Download
H-MAGMA, https://github.com/thewonlab/H-MAGMA
S-MultiXcan (version 0.6.5, git commit hash 78e3e5a17ecbd56169a93b905dfbbe92eafc6b63), https://github.com/hakyimlab/MetaXcan
GWAS-PW (version 0.21), https://github.com/joepickrell/gwas-pw
fgwas (version 0.3.6), https://github.com/joepickrell/fgwas
LDproxy (version 4.1.0), https://ldlink.nci.nih.gov/?tab=ldproxy

Additionally, the Supplementary Methods include software used previously for studies that constitute the meta-analysis but were not used in this study. These include:
Minimac3 (version 2.0.1), https://genome.sph.umich.edu/wiki/Minimac3#Download
IMPUTE2, https://mathgen.stats.ox.ac.uk/impute/impute_v2.html
BCFTools, https://samtools.github.io/bcftools/bcftools.html
Bolt-LMM, https://alkesgroup.broadinstitute.org/BOLT-LMM/

For manuscripts utilizing custom algorithms or software that are central to the research but not yet described in published literature, software must be made available to editors and reviewers. We strongly encourage code deposition in a community repository (e.g. GitHub). See the Nature Research guidelines for submitting code & software for further information.

Data

Policy information about availability of data

All manuscripts must include a data availability statement. This statement should provide the following information, where applicable:

- Accession codes, unique identifiers, or web links for publicly available datasets
- A list of figures that have associated raw data
- A description of any restrictions on data availability

The prior meta-analysis summary statistics are available via dbGaP accession number phs001532.v1.p1 [http://www.ncbi.nlm.nih.gov/projects/gap/cgi-bin/study.cgi?study_id=phs001532.v1.p1]. The summary statistics generated from the current study are included under version 2 of this dbGaP study with accession number phs001532.v2.p1 [http://www.ncbi.nlm.nih.gov/projects/gap/cgi-bin/study.cgi?study_id=phs001532.v2.p1]. These summary statistics are also available upon reasonable request to the corresponding author (D.B.H.). Individual-level genotype and phenotype data for many of the contributing studies are available via dbGaP, as outlined in the study descriptions in the Supplementary Methods The dbGaP accession numbers for these studies are phs000092.v1.p1 [http://www.ncbi.nlm.nih.gov/projects/gap/cgi-bin/study.cgi?study_id=phs000092.v1.p1], phs000404.v1.p1 [http://www.ncbi.nlm.nih.gov/projects/gap/cgi-bin/study.cgi?study_id=phs000404.v1.p1], phs000095.v2.p1 [http://www.ncbi.nlm.nih.gov/projects/gap/cgi-bin/study.cgi?study_id=phs000095.v2.p1], phs000765.v1.p2 [http://www.ncbi.nlm.nih.gov/projects/gap/cgi-bin/study.cgi?study_id=phs000765.v1.p2], phs000093.v2.p2 [http://www.ncbi.nlm.nih.gov/projects/gap/cgi-bin/study.cgi?study_id=phs000093.v2.p2], phs000170.v2.p1 [http://www.ncbi.nlm.nih.gov/projects/gap/cgi-bin/study.cgi?study_id=phs000170.v2.p1], phs000021.v3.p2 [http://www.ncbi.nlm.nih.gov/projects/gap/cgi-bin/study.cgi?study_id=phs000021.v3.p2], phs000167.v1.p1 [http://www.ncbi.nlm.nih.gov/projects/gap/cgi-bin/study.cgi?study_id=phs000167.v1.p1], phs000286.v3.p1 [http://www.ncbi.nlm.nih.gov/projects/gap/cgi-bin/study.cgi?study_id=phs000286.v3.p1], phs000090.v1.p1 [http://www.ncbi.nlm.nih.gov/projects/gap/cgi-bin/study.cgi?study_id=phs000090.v1.p1], phs000277.v1.p1 [http://www.ncbi.nlm.nih.gov/projects/gap/cgi-bin/study.cgi?study_id=phs000277.v1.p1], phs000092.v1.p1 [http://www.ncbi.nlm.nih.gov/projects/gap/cgi-bin/study.cgi?study_id=phs000092.v1.p1], and phs000404.v1.p1 [http://www.ncbi.nlm.nih.gov/projects/gap/cgi-bin/study.cgi?study_id=phs000404.v1.p1]. 1000 Genomes Phase 3 reference panel data are available at ftp://ftp.1000genomes.ebi.ac.uk/vol1/ftp/release/20130502/. GSCAN summary statistics are available at https://genome.psych.umn.edu/index.php/GSCAN. GWAS summary statistics from LD Hub are available at http://ldsc.broadinstitute.org/gwashare/. LDSC EUR LD scores are available at ftp://atguftp.mgh.harvard.edu/brendan/1k_eur_r2_hm3snps_se_weights.RDS. UK Biobank GWAS summary statistics are available at http://www.nealelab.is/uk-biobank/. LD blocks used with GWAS-PW are available at https://bitbucket.org/nygcresearch/ldetect-data/src/master/EUR/fourier_ls-all.bed. BrainSeq Consortium cis-eQTL summary statistics are available at http://eqtl.brainseq.org/phase2/eqtl/.

Field-specific reporting

Please select the one below that is the best fit for your research. If you are not sure, read the appropriate sections before making your selection.

☒ Life sciences ☐ Behavioural & social sciences ☐ Ecological, evolutionary & environmental sciences

For a reference copy of the document with all sections, see nature.com/documents/nr-reporting-summary-flat.pdf

Life sciences study design

All studies must disclose on these points even when the disclosure is negative.

Sample size	Sample sizes were determined based on the availability of data. A goal of this study was to amass as large of a sample size as feasible from pre-existing studies to identify genetic variants associated with nicotine dependence. This work is the largest nicotine dependence genome-wide association study to date.
Data exclusions	Samples and genetic variants were excluded from final analyses based on pre-established standard quality control procedures. Participants were removed due to missing rate >3%, sample duplication (identity-by-state >90%), first-degree relatedness (identity-by-descent >40%), gender discordance (Fst <0.2 for chromosome X single nucleotide polymorphisms (SNPs) to confirm females and Fst >0.8 to confirm males), excessive homozygosity (Fst >0.5 or Fst <-0.2), or chromosomal anomalies. SNPs were removed due to missing rate >3% or Hardy-Weinberg equilibrium P<1×10-4. Studies with a differing quality control process are noted in the Supplementary Methods.
Replication	Due to a lack of available datasets of comparable sample size for nicotine dependence, we relied on the heaviness of smoking index (HSI) that is available in the UK Biobank (N=33,791 biologically independent samples) for a single independent testing of our genome-wide significant FTND-based (Fagerstrom Test for Nicotine Dependence) GWAS meta-analysis findings. The UK Biobank collected data on two of the 6 FTND items (cigarettes per day and time to first cigarette in the morning) among current smokers. These two items comprise the HSI, which has historically been considered a suitable proxy for the full-scale FTND. Of the two novel genome-wide significant loci from the meta-analysis, one was consistently associated (i.e., successful independent test), but the other was not associated (i.e., unsuccessful independent test), likely reflecting ND features not captured by the HSI. This discrepancy is further discussed in the Discussion section.
Randomization	Samples were allocated into three nicotine dependence categories (mild, moderate, severe) based on FTND scores (described in the supplementary methods). Covariates (age, sex, etc.) were controlled for through inclusion as explanatory variables in the regression models for association testing. The supplementary methods describe the specific covariates used by each contributing study.
Blinding	Primary data collection was not a direct component of this work. Blinding is not relevant to this study as it is a genome-wide association study meta-analysis.

Reporting for specific materials, systems and methods

We require information from authors about some types of materials, experimental systems and methods used in many studies. Here, indicate whether each material, system or method listed is relevant to your study. If you are not sure if a list item applies to your research, read the appropriate section before selecting a response.

Materials & experimental systems

n/a	Involved in the study
☒	☐ Antibodies
☒	☐ Eukaryotic cell lines
☒	☐ Palaeontology and archaeology
☒	☐ Animals and other organisms
☐	☒ Human research participants
☒	☐ Clinical data
☒	☐ Dual use research of concern

Methods

n/a	Involved in the study
☒	☐ ChIP-seq
☒	☐ Flow cytometry
☒	☐ MRI-based neuroimaging

Human research participants

Policy information about studies involving human research participants

Population characteristics	Population characteristics for each of the 23 studies comprising the meta-analysis are detailed in Supplementary Table 2.
Recruitment	We did not do any recruitment as of part of the current study. Analyses were all performed on pre-existing data. Descriptions of recruitment strategies that generated the pre-existing data are described in the Supplementary Methods.
Ethics oversight	The meta-analysis work to combine results from the 23 studies was approved by the RTI International Institutional Review Board. The 23 studies that generated the summary statistics used in the meta-analysis had their study protocols approved by IRBs at their respective sites.

Note that full information on the approval of the study protocol must also be provided in the manuscript.
